# Postoperative analgesia for gynecological laparoscopy

**DOI:** 10.4103/1658-354X.57883

**Published:** 2009

**Authors:** Ben Gibbison, Stephen Michael Kinsella

**Affiliations:** *Department of Anesthesia, St. Michael's Hospital, Southwell St. Bristol, UK*

**Keywords:** *Analgesia*, *postoperative*, *laparoscopy*

## Abstract

Gynecological laparoscopy is a commonly performed procedure. Providing anesthesia for this can present a challenge, particularly in the day surgery population. Poor analgesia, nausea, and vomiting can cause distress to the patient and increased cost for the health system, because of overnight admission. In this review we discuss anesthetic and analgesic techniques for day-case gynecological laparoscopy. The principles include multimodal analgesia, the use of the oral route wherever possible, and the contribution of the surgeon.

## INTRODUCTION

Laparoscopy was first performed about a century ago, but came into more routine practice around 50 years ago.[1] This was initially confined to gynecological diagnostic procedures and sterilization. The rapid advances that have occurred in surgical procedures date from developments by general surgeons. Appendectomy was first performed 30 years ago and cholecystectomy 20 years ago. Using these techniques, gynecological laparoscopical surgery has developed recently, and is used for ovarian surgery, laparoscopically-assisted vaginal hysterectomy (LAVH), and LAVH with radical hysterectomy. For diagnostic surgery, there is a clear reduction in operative trauma with laparoscopy compared to laparotomy. However, for a more extensive surgery such as this, the comparative reduction in trauma will be less. Other benefits of laparoscopic surgery include reduced hospital stay, as also improving cosmetic results and patient satisfaction.

This review will focus on day-case laparoscopy. It is essential that the analgesia provided is sufficient for the patient to mobilize by the evening of surgery and control the pain which may persist for several days after the operation. The pattern of pain may change over this period, with pain in different sites becoming more or less prominent.[2] Analgesia provision is particularly important in the UK, where day-case surgery means that the unit closes in the evening, unlike in the USA where it means staying for up to 23 hours. Anesthetic and surgical techniques must therefore take into consideration pain that occurs outside the direct observation of the medical team. Steps must also be taken to minimize side effects such as nausea, vomiting, and dizziness. A suggested target for overnight admission due to pain or nausea is ≤1%.[3]

## MECHANISMS OF PAIN AFTER LAPAROSCOPY

Local pain will be associated with incisions for the operative ports. Lower abdominal pain may depend on the extent of intraperitoneal manipulation during diagnostic laparoscopy. Sterilization operations cause ischemia or damage to the fallopian tubes and are generally more painful than simple diagnostic procedures, with clips generally causing less pain than other techniques to occlude the tubes.

Upper abdominal, shoulder tip, and postural high back pain after laparoscopy are likely to be caused by gas retained in the peritoneal cavity. Carbon dioxide is usually used to expand the abdomen to allow surgical visualization. Although it is a soluble gas in comparison to oxygen and nitrogen, it can take up to two days to be absorbed from the peritoneal cavity. Pain from the residual gas is of delayed onset and may present once the patient has gone home. Hohlrieder *et al*. found that the worst pain after gynecological laparoscopic surgery was felt in the shoulder in 1% of the patients, two hours after surgery, but in 70% of the patients 24 hours after surgery.[4] Stanley *et al*. found that the pain attributed to intraperitoneal gas was as frequent as abdominal wall pain at 24 hours, but declined markedly by 48 hours, along with a corresponding reduction in the retained gas shown on X-ray.[5]

## ANALGESIC DRUGS

There are many trials of individual drugs versus placebo, but relatively few direct comparisons between drugs. A systematic review has allowed the production of comparative Tables of analgesic strength.[6] The results are expressed as Number Needed to Treat (NNT). Paracetamol 1 g has an NNT of 4.6 to reduce moderate-to-severe pain by 50%, but this is reduced to 1.9 for combination paracetamol 1 g/codeine 60 mg. The NNT for intramuscular morphine 10 mg is 2.9. The most effective non-selective non-steroidal anti-inflammatory drug (NSAID) is diclofenac 100 mg, with an NNT of 1.8.[6] Specific COX-2 inhibitors appear to be more efficacious, although there have been concerns over the increased cost and increased risk of thrombotic events with their use. Most anesthetists use multimodal analgesia; that is, the use of multiple drugs to optimize the analgesia, while avoiding side effects from large doses of individual agents. We currently use a variety of drugs and anesthetic interventions that have been shown to be effective in themselves [Table 1]. The overall ‘recipe,’ however, is rarely formally assessed.

**Table 1:** Day surgery analgesia guidelines used at St. Michael's Hospital, Bristol, UK*

	Options/comments
Oral premedication (2 hours preoperative)	
Paracetamol 1 g	Soluble paracetamol if difficulty in swallowing tablets
Meloxicam 15 mg (7.5 mg for age > 70) + small amount of water to help swallow	Diclofenac enteric coated 100 mg (or 50 mg)
Intraoperative	
(if no premedication has been given, intravenous paracetamol 1 g and parenteral NSAID at induction)	
Fentanyl 200 μg	Adjust dose according to population; reduce/omit if high risk of PONV
Morphine 10 mg	Adjust dose according to population; omit if medium risk of PONV
Sterilization operation-local anesthetic into mesosalpinx 0.25% bupivacaine 2 mg/kg divided between peritoneum and wounds gas drain	Ensure maximum amount of gas vented before port removal
Postoperative	
For severe pain	
PACU - intravenous fentanyl 20 μg increments up to 3 minutes	
Step-down ward-intramuscular morphine 7.5-10 mg	
For moderate/mild pain	
Paracetamol 1 g - check time elapsed since first dose	
Codeine 30-60 mg	Oral morphine solution - check local drug dispensing guidelines for dose and frequency
NSAID - check time elapsed since first dose tramadol-if NSAID contraindicated

* See column 1; NSAID - Non-steroidal anti-inflammatory drug; PONV - Post-operative nausea and vomiting; PACU - Post-anesthesia care unit = Recovery ward

## PREEMPTIVE ANALGESIA

Providing analgesia before the surgical stimulus should, according to theoretical principles, reduce the amount of pain felt after surgery compared to cases where analgesia has been given after the surgical stimulus. In clinical practice this has at best only a minor effect.[2] However, giving the analgesic drugs before the operation or early on during the operation will ensure that drug absorption and distribution to the effect site has occurred by the time the patient is waking after general anesthesia. Optimum analgesia given before waking will reduce the need for strong opioids in the Post-Anesthesia Care Unit (PACU; recovery ward). This is carried out with the aim of reducing side effects and improving patient satisfaction.

## ROUTES OF ADMINISTRATION

The oral route is acceptable for day-case surgery as the gastrointestinal function is generally not significantly affected (unless there is postoperative nausea and vomiting (PONV)). Tablets may be used for premedication as well as postoperatively once the patient can tolerate drinking. Starvation guidelines are often more stringently applied than necessary because of the difficulty in coordinating the last intake and the start of anesthesia. Although this is defensible in the case of food, because of the consequences of pulmonary aspiration of undigested food, it is less rational for fluid intake. Water, isotonic drinks, and clear fruit juice are cleared from the stomach within two hours.

Suppositories are often given during general anesthesia, in which case it is sensible to do this before surgery rather than at the end to allow time for drug absorption and distribution. Drugs that have to be given intramuscularly such as prochlorperazine should preferably be given during general anesthesia, for patient comfort.

Patients will often have an intravenous cannula in place for several hours after surgery, and therefore the intravenous route is also an option after surgery, especially in the presence of PONV.

## PARACETAMOL (ACETAMINOPHEN)

Paracetamol is on the bottom step of the World Health Organization's pain ladder. It is useful for mild-to-moderate pain and has a morphine-sparing effect of up to 20% when used for more severe pain.[7,8] It does not, however, reduce the incidence of morphine side effects. It is unclear whether paracetamol in combination with an NSAID confers any benefit on the NSAID alone.[9,10] However, paracetamol is cheap and has few side effects and interactions. It would therefore seem sensible to use it. One interesting interaction is that with 5-HT_3_ receptor antagonists. Although the mechanism of paracetamol analgesia is complex and not yet fully elucidated, part of its effect is mediated via a central serotonergic mechanism,[11] which may be antagonized by 5-HT_3_ receptor antagonists such as granisetron.[12] This has only been shown *in vivo*; 5-HT_3_ receptor antagonists do not directly antagonize paracetamol *in vitro,* and thus an indirect mechanism has been postulated.

There is no absorption of paracetamol from the stomach, but there is a rapid uptake from the duodenum and small intestine. The optimum administration will therefore be when the stomach is empty. Peak plasma concentrations occur about 10 to 60 minutes after oral administration. It has been found to have a more variable onset when administered after minor surgery, possibly because of delayed gastric emptying after surgical stress.[13] The use of a starting dose for oral administration of paracetamol 2 g has been suggested in order to achieve a more rapid and reliable increase in concentration at the effect site. However, this reduces the number of postoperative doses that can be given in the first 24 hours.

Paracetamol may also be given as an intravenous dose of 1 g. This is well-tolerated and produces rapid peak plasma levels. The only disadvantage is the price, which is approximately 150 times the cost of tablets. However, intravenous paracetamol is convenient to use at induction of anesthesia if paracetamol premedication has been omitted.

## NON-STEROIDAL ANTI-INFLAMMATORY DRUGS

NSAIDs alone or in combination with paracetamol are unlikely to provide adequate analgesia in the early postoperative period by themselves, but may reduce the need for opioids.[2] Although the majority of studies show a benefit, this is not universal. However, they are widely used in day-case anesthesia. As mentioned above diclofenac 100 mg is the most efficacious of the non-selective NSAIDs (NNT 1.8). This is reduced to an NNT of 2.7 for 50 mg. Whatever may be the dose chosen, it is more efficacious than intramuscular morphine 10 mg (NNT 2.9). An analgesic regime based around an NSAID and supplemented by paracetamol and an opioid is likely to be the most effective for the perioperative period. This may then be simplified to a paracetamol-based regime, supplemented by an NSAID, when leaving the day surgery unit. This leads to good quality pain relief without the need to visit the patient's family doctor.[14]

There are a significant minority of patients in whom NSAIDs are relatively or absolutely contraindicated. Some anesthetists give them to patients with gastric sensitivity and asthma, but this should be as a result of a risk-benefit analysis and after discussion with the patient. Gastric side effects are not completely avoided by parenteral or rectal administration, and options that may be considered include the use of COX-2 selective NSAIDs (-oxicams or -coxibs), gastroprotective drugs (H_2_ antagonists, proton pump inhibitors or misoprostol), and slow-release formulations. However, it should be noted that these drugs have their own side effects (particularly the -coxib group of drugs and thrombotic events).

## OPIOIDS

Opioids feature at the top of the World Health Organization's pain ladder and are usually used as part of the day-case general anesthesia, although a completely opioid-free technique should possibly be considered for patients undergoing minor surgery at very high risk of PONV. Opioids may be given pre-, peri- and postoperatively and may be short- or long-acting.

Preoperative opioids have been trialed in day-case laparoscopic gynecological surgery. Both morphine[15] and controlled release oxycodone[16] given prior to induction have failed to reduce pain scores when compared to controls.

Morphine used perioperatively has a prolonged emetic effect, and the use of shorter acting synthetic opioids is more common. Claxton *et al*. found that fentanyl was associated with less PONV compared to morphine, and this benefit extended into the period when the patients went home.[17] However, patients in the fentanyl group required around two-and-a-half times the amount of supplementary oral analgesia, compared to those in the morphine group. Thus, if intraoperative morphine is used, consideration should be given to the prophylactic anti-emetics (see a little later in the text).

Tramadol is an atypical opioid with effects at the μ-opioid receptors and central noradrenergic and serotonergic pathways. It has less respiratory depression than morphine. When compared with ketorolac it provides consistently lower pain scores and an insignificant difference in PONV after laparoscopic sterilization.[18]

Postoperatively, intravenous opioids may be incrementally titrated by nurses in the PACU, according to a simple algorithm.[19]

Opioids also display well-documented interindividual variability. This can be a result of acquired or inherited differences. Genetic variability is seen in both the pharmacodynamics (i.e., the opioid receptors) and the pharmacokinetics (i.e., metabolism) of opioids.[20] A mutation in the μ-opioid receptor (A118G) exhibits greater binding and potency of β-endorphins to the receptor. This genetic polymorphism exists at a much higher rate in the South East Asian population (35-47%) compared to the Caucasian population (11.5%). Morphine requirements in the Asian population may therefore be expected to be less on average. Racial differences are much more complex than this. A study comparing the effects of morphine on the Chinese and Caucasians found that Caucasian individuals had a greater respiratory depression and a reduced ventilatory response to hypercapnia.[21] This is probably due to the increased clearance rates in the Chinese population. Pharmacogenetic differences in the cytochrome P450 (CYP450) enzymes may account for differences in the effects of codeine. Conversion of inactive codeine to active morphine by O-demethylation is by CYP2D6. This enzyme is absent in 7-10% of the Caucasian population and thus codeine is essentially ineffective in these patients. The converse of this is that some individuals are ultra-rapid metabolizers in whom morphine is produced rapidly. This can lead to opioid toxicity after a single dose of codeine. The incidence of ultra-rapid metabolism is particularly high in the Saudi Arabian population (21%) compared to the Caucasian British population (1-3%).

## ANALGESIC ADJUNCTS

Other drugs, outside the traditional analgesics have been trialed for postoperative analgesia. In a recent study from Finland,[22] the γ-aminobutyric acid (GABA) analog pregabalin was used for postoperative pain relief in day-case laparoscopic gynecological surgery. However, it failed to demonstrate any advantage over diazepam 5 mg (active control) in reducing postoperative morphine usage.

Ketamine given preoperatively demonstrates an analgesic benefit over that given postoperatively.[23] However, there is no evidence comparing ketamine against a conventional opiate/NSAID-based regime.

## LOCAL ANESTHESIA

Moiniche *et al*. performed a systematic review of local anesthetic techniques for laparoscopy.[24] They found that there was no statistically significant reduction in pain scores after infiltration into the port sites, although mesosalpinx block and other forms of direct application of local anesthetic to the fallopian tubes did reduce pain scores after laparoscopic sterilization. Although the mesosalpinx block needed some surgical training, the dose of the local anesthetic required was approximately only 2 ml per side, because of the precise application.[25] Other techniques included in the direct application group were local anesthetic on the Filshie clips (see a little later in the text) and dripping local anesthetic onto the fallopian tubes. Statistically, intraperitoneal local anesthetics reduced pain scores significantly, although the authors felt that a mean reduction of 13 mm on a visual analog score was clinically insignificant. All these studies used intraperitoneal bupivacaine. A study reported since then has compared ropivacaine 150 mg and bupivacaine 100 mg intraperitoneally[26] (the maximum dose of ropivacaine is higher than that of bupivacaine, due to its reduced cardiac toxicity). This found that ropivacaine reduced morphine usage in the first 24 hours approximately four-fold. Both the local anesthetics reduced PONV. This may be clinically significant.

Coating Filshie clips with local anesthetic has also been tried; either as aqueous lignocaine[27] or as a gel.[28] Pain scores were lower in both trials when lignocaine was applied to the clips. However, this was only significant one hour postoperatively. No trials have ever used longer acting bupivacaine. Any maneuver that reduce pain scores on waking up from general anesthesia will improve the patient's experience and the risk of PONV (see a little further in the text).

## GAS DRAIN

Alexander and Hull studied the effectiveness of a gas drain in pain reduction after laparoscopy.[29] This consists of a suction catheter with end and side holes, which was fed through the trocar at the end of the operation. The trocar was removed around the catheter and a safety pin inserted through the catheter. The drain was removed after 6 hours. Median visual analog scores for gas-related pain were reduced up to and including the afternoon of the first postoperative day, and pain frequency was reduced up to and including the second postoperative day. A placebo-controlled, randomized trial from Australia used a blocked gas drain as a placebo. It showed that pain scores were significantly reduced for up to 72 hours postoperatively when a patent gas drain was present for four hours postoperatively. There was no associated morbidity in their group of 80 patients.[30] However, in a further study from the same group, Readman *et al*. found that a gas drain and intraperitoneal ropivacaine conferred benefits only for the first four hours.[31]

More simple methods for removing carbon dioxide at the end of laparoscopy have been employed. Phelps *et al*. showed that pain scores were approximately halved when gas was actively removed from the abdominal cavity by placing the patient in the Trendelenberg position and giving five manual inflation breaths. This was compared to a control group in which the abdomen was passively deflated via the cannula. The reduction in pain was maintained up to 48 hours post surgery. PONV was also reduced from 56% in the control group to 20% in the actively deflated group.[32]

## GAS WARMING

A recent meta-analysis has shown that warming and humidifying the insufflated carbon dioxide reduces pain significantly after laparoscopic surgery, up to three days following the operation.[33] However this has not been confirmed in gynecological laparoscopy.[34]

## ANTIEMETICS

About 20 to 30% of the surgical patients will experience postoperative nausea and vomiting (PONV) and about 1% of day surgical patients will be admitted overnight because of it.

Apfel *et al*. demonstrated that the risk factors for PONV are the female sex, non-smoking, history of PONV or motion sickness, and postoperative morphine.[35] The first factor will be present in all cases, the second and third can be assessed by the anesthetist, to determine the patient's risk, but only the fourth is within the control of the anesthetist. In a subsequent study Apfel *et al*. also found a dose-dependent effect of volatile anesthesia.[36]

The cost-effectiveness of prophylactic anti-emetics for all patients has been debated, especially for more expensive drugs. Scuderi *et al*. showed that PONV could be reduced by multimodal prophylaxis, including total intravenous anesthesia and three antiemetic agents.[37] However, patient satisfaction was the same with both multimodal and single-agent treatment. Standard doses of dexamethasone, ondansetron, and droperidol could be regarded as essentially equipotent and additive in their effect. A group from Spain calculated that a combination of ondansetron and droperidol was as effective as a combination of dexamethasone and droperidol, and a little less expensive. This study was published in 2003, since when the patent on ondansetron has expired, and costs may have reduced further. Intraoperative fluid may also play a part in the incidence of PONV. A randomized control trial by Magner *et al*. showed that increasing the intraoperative fluid prescription from 10 ml/kg to 30 ml/ kg, for gynecological laparoscopy, reduced the cumulative incidence of PONV over the 72-hour perioperative period from 27 to 6%.[38]

Low-dose propofol infusion has previously been used for rescue anti-emesis. However, a recent study in patients undergoing gynecological laparoscopy found that bolus doses of propofol 0.5 mg/kg administered at the end of surgery were effective in reducing PONV, when compared to placebo.[39] Lower doses than this were not effective.

Targeting individual patients with the risk stratification of Apfel *et al*. may allow a sensible compromise between ‘blanket’ treatment and no prophylaxis. It is probable that these interventions apply only to early PONV. Different factors apply to the occurrence of PONV beyond 24 hours.

## INFLUENCE OF THE ANESTHETIC TECHNIQUE

Hohlrieder *et al*. found that early pain, nausea, and 24-hour morphine consumption using patient-controlled analgesia was less in women who had their airway managed with a Proseal laryngeal mask airway (PLMA) compared to tracheal intubation.[4,40] Their explanation was that tracheal intubation activates pain centers, and this has a cross-over effect on surgical pain. Whatever the explanation, this study demonstrates that unexpected factors in the patient's management may have a bearing on the overall analgesic outcome. Concerns over the risk of aspiration when using an LMA have not been borne out, providing the patient has no history of gastroesophageal reflux.[41,42] The PLMA would seem to be the logical choice for laparoscopy as it allows separation of the respiratory and gastrointestinal tracts as well as allows controlled ventilation at higher seal pressures than the classic LMA.

The European Society of Regional Anesthesia and Pain Therapy produce evidence-based guidelines on the anesthetic technique for specific procedures.[43] However, the only laparoscopic procedure included at the moment is cholecystectomy, but gynecological procedures may probably be included in the future.

## POSTOPERATIVE ANALGESIA-IN THE PACU AND STEP DOWNWARD

For the immediate control of severe pain on waking after general anesthesia, intravenous opioids should be titrated to effect. Both fentanyl and morphine can be used. Morphine leads to a reduction of about three-and-a-half times the amount of supplementary oral analgesia used, compared to fentanyl, but causes about twice as much nausea and vomiting.[17]

We prescribe intramuscular morphine as escape analgesia for the step-down ward. The aim is to give this rarely, which will be true if the patients have had sufficient attention paid to analgesia at the earlier stages of the procedure. Other postoperative analgesia prescriptions are paracetamol, codeine, and diclofenac.

## ‘TAKE HOME’ ANALGESIA

A combination of paracetamol 1 g/codeine 60 mg four times a day, as well as an NSAID (for example diclofenac or ibuprofen) should be prescribed for five days.[14] It should be explained to the patients that these doses should be taken on a regular basis to start off with when they get home, so that severe pain that will be difficult to treat does not develop.

## CONCLUSION

There are a vast number of possible permutations to a routine analgesic and antiemetic regimen, as well as one tailored for patients with specific problems. Furthermore, drug availability and social, genetic and racial differences are further confounding factors. The key principles are to have a multimodal regimen, regular pain scoring, and encouraging patients to ask for analgesia. Patients' recovery to full function is enhanced by good quality postoperative analgesia.
